# Medical Images Segmentation for Lung Cancer Diagnosis Based on Deep Learning Architectures

**DOI:** 10.3390/diagnostics13030546

**Published:** 2023-02-02

**Authors:** Yahia Said, Ahmed A. Alsheikhy, Tawfeeq Shawly, Husam Lahza

**Affiliations:** 1 Department of Electrical Engineering, College of Engineering, Northern Border University, Arar 91431, Saudi Arabia; 2 Laboratory of Electronics and Microelectronics (LR99ES30), University of Monastir, Monastir 5019, Tunisia; 3 Department of Electrical Engineering, Faculty of Engineering at Rabigh, King Abdulaziz University, Jeddah 21589, Saudi Arabia; 4 Department of Information Technology, College of Computing and Information Technology, King Abdulaziz University, Jeddah 21589, Saudi Arabia

**Keywords:** lung cancer segmentation, lung cancer classification, medical images, deep learning, transformers

## Abstract

Lung cancer presents one of the leading causes of mortalities for people around the world. Lung image analysis and segmentation are one of the primary steps used for early diagnosis of cancer. Handcrafted medical imaging segmentation presents a very time-consuming task for radiation oncologists. To address this problem, we propose in this work to develop a full and entire system used for early diagnosis of lung cancer in CT scan imaging. The proposed lung cancer diagnosis system is composed of two main parts: the first part is used for segmentation developed on top of the UNETR network, and the second part is a classification part used to classify the output segmentation part, either benign or malignant, developed on top of the self-supervised network. The proposed system presents a powerful tool for early diagnosing and combatting lung cancer using 3D-input CT scan data. Extensive experiments have been performed to contribute to better segmentation and classification results. Training and testing experiments have been performed using the Decathlon dataset. Experimental results have been conducted to new state-of-the-art performances: segmentation accuracy of 97.83%, and 98.77% as classification accuracy. The proposed system presents a new powerful tool to use for early diagnosing and combatting lung cancer using 3D-input CT scan data.

## 1. Introduction

Cancer is becoming one of the most frequent causes that lead to deaths around the world. According to the latest statistics of global cancer statistics (GLOBOCAN) [1], there are 19.3 million new cancer cases around the world. In 2020, 9.96 million people died from cancer. Lung cancer segmentation presents one of the most important research fields, and many studies have been elaborated. Numerous cancer treatment techniques have been developed to control malignant tumors and to enhance life quality for cancer patients, in addition to surgical restriction [2], including chemotherapy [3], radiation [4], thermotherapy [5], and immunotherapy [6].

Radiation therapy (RT) has made significant strides recently and is an essential component of lung cancer control [7]. The achievement of RT relies on precisely irradiating the tumor targets, protecting the organs at risk (OARs), and preventing consequences from radiotherapy. To deliver the prescribed dose to the gross tumor volume (GTV), it is crucial to segment GTV and OARs during RT treatment. Precise planning to manually segment the GTV and OARs by radiation oncologists presents a difficult task to perform. This could cause considerable RT treatment delays and low survival rates, especially in clinics with insufficient funding. Proficiency in manual segmentation also depends on radiation oncologists’ expertise. There can still be inconsistencies in the segmentation for both intra- and interobservers, even if they segmented the GTV and OARs following the same rules. On the other hand, the automatic segmentation method may result in accurate and efficient diagnoses. Several image segmentation methods have been introduced recently, leading to more precise and effective image segmentation for clinical diagnosis and treatment [8]. The diagnosis of lung cancer at an early stage and the monitoring of lung cancer throughout therapy need the use of medical imaging technologies. Lung cancer detection has been thoroughly researched using a variety of medical imaging modalities, including chest X-rays, magnetic resonance imaging, positron emission tomography, computed tomography, and molecular imaging methods. Lung cancer presents the first cause and highest morbidity and fatality rates in different countries worldwide [9,10].

Non-small-cell lung carcinoma (NSCLS) accounts for about 85–88% of cases of lung cancer, while small-cell lung cancer (SCLC) accounts for 12–15% of cases [11]. Lung cancer is invasive and heterogeneous, making early detection and treatment crucial to improving the five-year survival rate overall [12]. The detection of lung nodules has been thoroughly studied over the past decade using a variety of medical imaging techniques, including chest X-ray, positron emission tomography (PET), magnetic resonance imaging (MRI), computed tomography (CT), low-dose CT (LDCT), and chest radiograph (CRG).

It is crucial to pay more attention to the medical field and, especially, to build new systems used for early cancer diagnosis in order to limit its mortality rate. The segmentation accuracy is a key consideration when using lung cancer segmentation results. Radiologists typically perform segmentation manually, but manual segmentation occasionally produces unreliable results due to interobserver variability due to inconsistency. To address this problem, an automatic segmentation system of CT scan lung cancer images is highly relevant.

Deep-learning-based models have recently demonstrated great performance in various computer vision and artificial intelligence fields. The use of deep learning in the medical field has witnessed significant development, especially when applied to organ cancer segmentation. Deep-learning-based models have demonstrated more outstanding capabilities in the auto-segmentation of medical images. Deep learning models autonomously learn feature representation and then use the high-dimensional abstraction they have to complete segmentation tasks without any manual intervention. One of the most frequent issues in applying deep learning architectures is using massive datasets. Small datasets do not contribute to better results when applying deep learning models. Compared to other fields, expanding the number of datasets, including medical images, is generally challenging. This is because obtaining medical imaging is expensive, and patients’ privacy needs protection. Transfer learning of a pre-trained model [13], data augmentation [14], and artificial dataset generation utilizing generative adversarial networks (GANs) have been proposed [15] to overcome the need for large-scale datasets in the training stage. Applying deep learning to the healthcare field has solved other health issues by using deep learning for classification, detection, or segmentation. Automatically detecting and diagnosing cancer in CT scans with deep learning is extremely important to prevent cancer from advancing to the metastasizing stages.

Medical image segmentation presents a fundamental axis in computer vision. This task presents one of the most critical and challenging parts of image processing. Medical image segmentation has succeeded dramatically, especially after the appearance of deep learning, and made tremendous advances, especially after the arrival of deep learning and deep convolutional network architectures.

Lung cancer is the second most common type of cancer, representing 11.4% of all new cases. Meanwhile, it dominates cancer-related mortality globally, accounting for 18.0% of all cancer-related deaths [1].

Based on the above, designing an early lung cancer diagnosis system is extremely important to save lives and prevent metastasized stages.

The target is often segmented using conventional automatic segmentation algorithms based on the shallow properties of the image, such as the grayscale, texture, gradient, etc. Many works on lung segmentation have utilized traditional image processing methods, such as thresholding, edge detection, and clustering [16].

Deep learning as a subfield of artificial intelligence is getting more attention in image automatic segmentation due to significant advancements in computing methodologies and data accumulation [17]. Deep learning, such as convolutional neural networks, fully convolutional networks (FCNs) [18], and U-Net, can represent more complex phenomena, in addition to continuing to increase the model depth by hierarchically extracting features from the input data via the hidden layers and repeatedly training the network with the input data [19]. Further, these architectures have been widely used in different studies to solve the problem of image segmentation. In the medical field, other works have been proposed to address the issue of lung cancer detection, as it presents the second highest cancer mortality cause around the world [1].

In [20], Nishio et al. proposed a new lung cancer segmentation system using deep learning techniques. Their study aimed to create and assess a segmentation method for lung cancer using transfer learning and a pre-trained model. An artificial dataset produced by a generative adversarial network served as the basis for the pre-trained model’s construction. The authors obtained a good Dice similarity coefficient (DSC) for the NSCLC radio genomics dataset in this study. In [21], Liu et al. proposed a review of the different methods used to address the lung cancer segmentation issue. They also compared deep-learning-based segmentation techniques and the atlas method [22].

Cancer is a potentially fatal disease that requires early diagnosis to improve patient survival rates. Medical imaging is time-consuming and requires a high level of knowledge to manually detect, segment, and classify cancer in several organs (such as the breast, brain, lung, and skin). In [23], Wang et al. examined the most recent developments, difficulties, and potential paths for future research in deep learning approaches for lung cancer and pulmonary nodule identification. In [24], Ali et al. review current deep learning segmentation and classification techniques for multi-organ cancer diagnosis and outline potential future obstacles.

Currently, a reliable technique to precisely identify nodules from lung imaging is 3D-based segmentation. These qualities have been included in numerous strategies. Tomography scans were used by Paing et al. [25] to create a completely automated and improved random forest classification method for lung nodules. The borders are made better by using a 3D-chain code method. A 3D CNN technique was developed in [26] for the automatic diagnosis of lung cancer, and it produced effective results, with a recall of 99.6% and an AUC of 0.913%. To assess performance, the model was trained using the LIDC-IDRI standard dataset. Different studies have proved that integrating deep-learning-based techniques has widely improved medical imaging segmentation results [27].

The network for volumetric segmentation learns from sparsely labeled volumetric data. Two intriguing applications for this technique have been proposed: a fully automated and a semi-automated arrangement. The proposed network replaces all 2D operations with their equivalent 3D functions; the 3D-U-Net architecture was extended from the prior U-Net architecture by Ronneberger et al. [19]. Some of the state-of-the-art works have used 3D-based data and architectures to segment lung cancer. The majority of these works proved that the use of 3D data achieves more promising results than 2D-based data. In [28], nodule detection and classification methods are proposed. This study discussed an automated method of detection and classification to assist radiologists in diagnosing the disease. A high false-positive rate was achieved in these systems, which could result in the wrong diagnosis. To address this problem, a new 3D-based detection and classification technique was proposed for nodule lung cancer detection. In [29], the authors proposed to create and verify a deep-learning-(DL)-based model and evaluated its capability to identify lung cancer on chest radiographs. The developed deep learning model achieved a sensitivity rate of 0.73. Planning radiation therapy requires the precise identification of lung tumors. Segmenting the cancer in computed tomography (CT) images is complicated and complex because of the low contrast of the lung tumor. In [30], authors proposed a deep-leaning-based architecture for lung tumor segmentation. This study efficiently segments the cancerous lung area from the surrounding chest region using U-Net and the channel attention module (CAM). Compared to state-of-the-art models, the developed model, SegChaNet, achieved better results in learning the dense features of lung abnormalities.

Various works have been proposed in the literature to address the problem of lung cancer segmentation in medical imaging. Still, few of them offered a complete system that performs segmentation and diagnosis to classify the lung tumor as either malignant or benign.

This work aims to develop a new CT scan lung cancer segmentation system. The segmentation part of the proposed system was performed based on the UNETR neural network [31], which consists of a combination of U-Net and transformers. To ensure a full system used for early diagnosis of lung cancer, the proposed work presents a second part based on a classification head, which is performed on top of the Self-Supervised Classification Network [32]. After that, the segmentation results will be processed to be classified as either benign or malignant pulmonary nodules.

The proposed work presents various additional advantages compared to other state-of-the-art works, as it presents a full system for lung cancer segmentation and a diagnosis stage via a classification head. The majority of the state-of-the-art works focus on a single task, either a classification or segmentation task. The proposed work combines the two tasks to develop a full system used for early prediction of lung cancer tumor presence. The proposed system will highly contribute to the early diagnosis of lung cancer and avoiding metastasized situations. The proposed system will also assist doctors and experts in planning radiation therapy.

The main contributions of the proposed work are the following:

- Proposing an automatic lung cancer detection system based on the analyses of 3D CT scan images.
- Proposing the combination of a segmentation network followed by a classification network to predict lung cancer cases
- Proposing the use of a transformers-based segmentation network to collect more relevant features from the 3D input data
- Proposing the use of a self-supervised network for the classification of the segmented images
- Validating the performance of the proposed system on a publicly available dataset

The remainder of this paper is the following: Section 2 details the different parts of the developed system used for the early diagnosis of lung cancer. Section 3 details all the experiments conducted to contribute to the developed system. Discussion is provided in Section 4, and Section 5 concludes the paper.

## 2. Materials and Methods

### 2.1. Problem Statement

The analysis of image characteristics of lung nodules on CT images is crucial for computer-aided detection systems of lung cancer in order to distinguish between benign and malignant nodules. It is increasingly important to build an automatic segmentation system for early lung cancer diagnosis. Building such a system using classical methodologies is extremely difficult.

During the last few years, deep-learning-based architectures have gained significant attention and have undergone huge development to be used in the field of healthcare. Therefore, this study aimed to create a robust and precise 3D-segmentation approach using deep learning for lung cancer.

### 2.2. Research Objectives

The main aim of the proposed study is to build an early diagnosis system for lung cancer. The proposed system is mainly divided into two main parts: segmentation head and classification head used for further CT scan lung cancer classification.

### 2.3. Segmentation Head

The segmentation part is basically developed on top of a combination of transformers and U-Net networks. This architecture was proposed in [31] and named “UNETR”. The UNETR network uses a transformer as the encoder to effectively capture the global multiscale information and learn sequence representations of the input volume. The encoder and decoder are designed using the well-established “U-shaped” network architecture. To calculate the final semantic segmentation output, the transformer encoder is directly connected to a decoder via skip connections at various resolutions. In UNETR, the encoder and decoder are coupled by skip connections in a contracting–expanding pattern using a stack of transformers as the encoder. The transformers were widely used in the natural language processing field. Transformers operate using a 1D sequence of input. Similar to transformers, UNETR creates from 3D input data a 1D sequence. The 3D input data are x $\epsilon \;R^{H\;\times \;W\;\times \;H\;\times \;C}$; the input data present a resolution of (H, W, D, C). C presents the number of channels that are divided into homogeneous, non-overlapping, flattened patches. $x_{v}$ $\epsilon \;R^{N\;\times \;\left(P^{3}\;.\;C\right)}$, where each patch’s resolution is indicated by (P, P, P), N = (H × W × D)/P3, denotes the sequence length. Then, the patches are projected using a linear layer into a K-dimensional embedding space that is constant across all transformer levels. To be able to maintain the retrieved patches’ spatial information, a 1D learnable, positional, embedding Epos is added to the architecture. E∈$\;R^{\left(P^{3}\;.\;C\right)\times \;K}$ is according to Equation (1).

(1) $$z0=[x_{v}^{1}E,\;x_{v}^{2}E,\;\ldots ,\;x_{v}^{N}E]\;E_{\mathrm{pos}}$$

Following the embedding layer, a stack of transformer blocks is applied, composed of multi-head self-attention (MSA) and multilayer perceptron (MLP) sublayers. MSA and MLP are defined as Equations (2) and (3), respectively.

(2) $$Z_{i}^{\prime }=\mathrm{MSA}\left(\mathrm{Norm}\left(\mathrm{zi}-1\right)\right)+\mathrm{zi}-1,\;i=1\ldots L$$

(3) $$Zi=\mathrm{MLP}(\mathrm{Norm}(Z_{i}^{\prime }))+Z_{i}^{\prime },\;i=1\ldots L$$

The norm denotes layer normalization. Two linear layers with GELU activation functions compose the MLP, along with an intermediate block identifier i, and the number of transformer layers is L. MSA’s self-attention sublayer (SA sublayer) consists of n parallel SA heads. A parameterized function called the SA block, in particular, learns the mapping between a query (q) and the representations of the corresponding key (k) and value (v) in a sequence z $\epsilon \;R^{N\;\times \;K}$. The attention weights (A) are calculated by comparing two entries in z and their key-value pairs, as in Equation (4).

(4) $$A\;=\;\mathrm{Softmax}\frac{QK^{T}}{\sqrt{K_{h}}}$$

where K_h_ = K/n serves as a scaling factor to keep the several parameters that affect a constant value, the key’s values, which uses the calculated attention weights. The output of SA layers is calculated as in Equation (5) for v values in the sequence z.

(5) SA(z) = Av

Here, v represents the values in the input sequence, and a scaling factor, K_h_ = K/n, is used. Additionally, the MSA’s output is calculated as in Equation (6).

**Figure 1:**
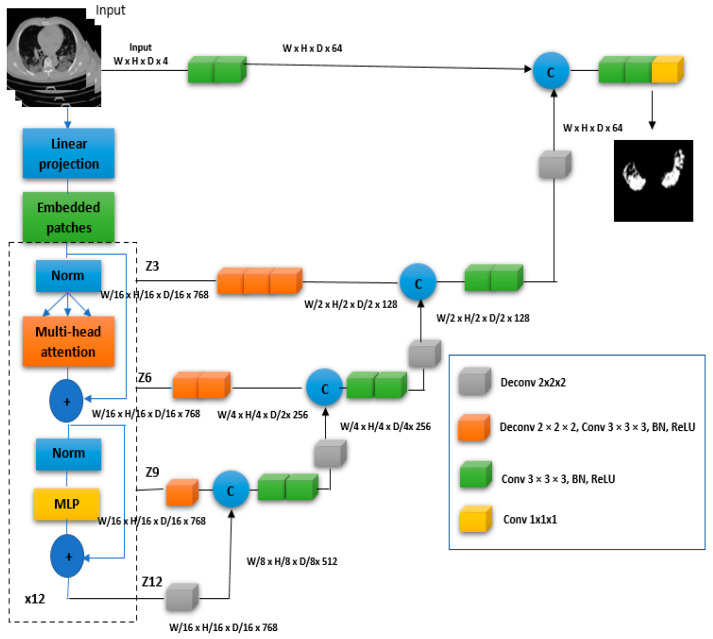
The UNETR architecture is used as a segmentation head.

(6) MSA(z) = [SA_1_(z); SA_2_(z); …; SA_n_(z)] W_msa_

where W_msa_ reflects the weights of the multiple trainable parameters. Figure 1 provides a detailed architecture of the UNETR architecture used for lung cancer segmentation.

### 2.4. Classification Head

In order to ensure an early diagnosis of lung cancer, the segmentation output will be fed into the classification head to classify input data as either benign or malignant. The “self-supervised neural network” [32] was adopted to perform the classification process for the proposed diagnosis system. This architecture presents an innovative end-to-end, self-supervised, classification learning technique. Self-Classifier optimizes for same-class prediction of two enhanced perspectives of the same sample, simultaneously learning labels and representations in a single-stage end-to-end process. A mathematically motivated variation of the cross-entropy loss with a uniform prior asserted on the projected labels is used to ensure non-degenerate solutions.

**Figure 2:**
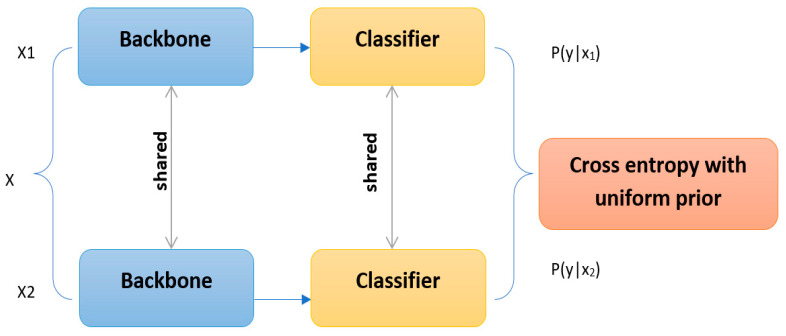
The self-supervised neural network architecture is used as the classification head.

Over the past few years, interest in self-supervised visual representation learning has grown [33]. The fundamental goal is to define and complete a pretext task so that representations with semantic meaning can be learned without needing human-annotated labels. It is possible to learn meaningful representations without any human-annotated labels. Later, the learned representations are applied to subsequent tasks by finetuning of a smaller dataset. Modern self-supervised models are built on the contrastive learning mind. These methods minimize the similarity between various images under different situations, while simultaneously maximizing the similarity between two alternative augmentations of the same image. The self-supervised neural network provides a classification-based pretext task whose target is, in this instance, closely related to the ultimate purpose. An unsupervised classifier (Self-Classifier) was designed to categorize two alternative augmentations of the same image similarly, while only knowing the number of classes, C. In reality, a task like this is prone to degenerate solutions, where every sample is put in the same class. The self-supervised neural network architecture is presented in Figure 2.

To address this problem, asserting a uniform prior on the common cross-entropy loss function to prevent them from making an answer that evenly divides the data presents the best option. In fact, this architecture demonstrates that degenerating solutions are no longer included in the set of optimal solutions. This architecture combines deep unsupervised clustering architectures [34] and contrastive learning [35]. The self-supervised architecture uses minibatch SGD to learn representations and cluster labels in a single-stage end-to-end process. This model presents a straightforward, efficient single-stage, end-to-end, self-supervised, classification and representation learning method. No pre-training, expectation-maximization technique, pseudo-labeling, or external clustering is necessary with this method.

Let us use the symbols *x*_1_ and *x*_2_ to represent two distinct augmented views of the identical image sample *x*. The main objective for this architecture is to learn a classifier *y* = f(*x_i_*) ϵ [*C*], where *C* is the specified number of classes that can classify two different views of the same sample. The following cross-entropy loss should be minimized as a naive solution to this problem:

(7) $$l\left(x_{1},x_{2}\right)=\sum_{y\epsilon \left[c\right]}P(y|x_{1})\;\log\;P(y|x_{2})$$

where *p*(*y*|*x*) is the row softmax of the matrix of logits S generated by our model (backbone + classifier) for all classes (columns) and batch samples (rows). The network predicts a constant y value independent of the *x*. Therefore, attempts to reduce Equation (7) without further regularization will quickly converge to this degenerate solution. To fix this, we suggest applying the Bayes and total probability laws, resulting in the following:

(8) $$P\left(y|x_{2}\right)=\frac{p\left(y\right)\;p\left(y|x_{2}\right)}{p\left(x_{2}\right)}=\frac{p\left(y\right)\;p\left(x_{2}|y\right)}{\sum_{y\epsilon \left[c\right]}P(x_{2}|y)p\left(y\right)}$$

(9) $$P\left(y|x_{1}\right)=\frac{p\left(y\right)\;p\left(y|x_{1}\right)}{p\left(y\right)}=\frac{p\left(y\right)\;p\left(y|x_{1}\right)}{\sum_{x_{1}\epsilon B_{1}}P(y|x_{1})p\left(x_{1}\right)}\;$$

where *B* is a collection of N samples (*B*_1_ are the batch’s first additions), S is the aforementioned matrix of logits, and *p*(*x*|*y*) is a softmax column.

In the self-supervised network, the authors assume that *p*(*x*_1_) is uniform (under the reasonable assumption that the training samples are equiprobable) and believe that *p*(*y*) has a uniform prior; the cross-entropy function will become Equation (10).

(10) $$l\left(x_{1},x_{2}\right)=-\sum_{y\epsilon \left[c\right]}\frac{\;p\left(x_{2}|y\right)}{\sum_{y}P(x_{2}|y)}\log\;(N/C)\frac{\;p\left(y|x_{1}\right)}{\sum_{y}p\left(y|x_{1}\right)}$$

In the proposed work, the self-supervised network was used to perform a complete lung cancer diagnosis and to classify segmented input data as either benign or malignant.

**Figure 3:**
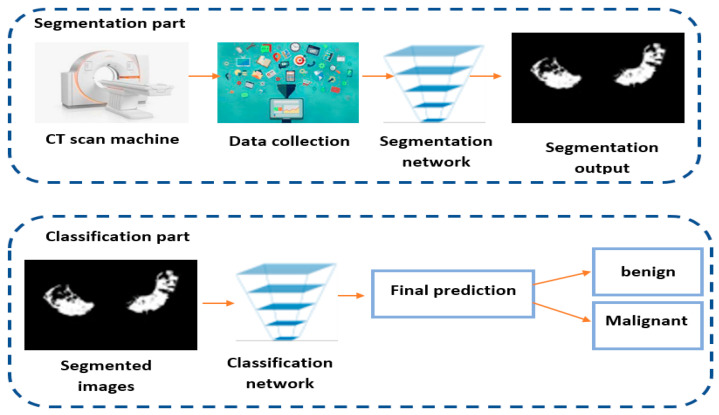
The entire architecture of the lung cancer diagnosis system.

A combination between UNETR and a self-supervised network will contribute to performing an early diagnosis of lung cancer. Figure 3 provides the central architecture of the diagnosis system.

## 3. Results

The main objective of this work is to develop a tool featuring segmentation and a classification system used to ensure an early diagnosis of lung cancer. The effectiveness of deep-learning-based methods for lung cancer classification and detection is largely evaluated by lung imaging datasets. The Decathlon dataset [36] was used for training and evaluating the proposed model. The Decathlon challenge (http://medicaldecathlon.com (accessed on 15 December 2022)) was organized to offer a complete, open-source benchmark for general-purpose algorithmic validation and testing that addressed several segmentation tasks. The Decathlon lung dataset (Task06), one of several segmentation datasets included in Decathlon, served as the study’s training and validation sets. The Decathlon lung dataset consists of 96 sets of segmented 3D CT scans. The dataset was divided into two subsets: train with 64 input 3D volumes and test with 32 3D volumes.

In the proposed work, the diagnosis system consists of a combination of two parts: a segmentation head and a classification head. The input data of the segmentation part are a 3D CT scan, which the UNETR network will process to perform a semantic segmentation as an output. The output of the segmentation part will be fed into the classification part, which is built on top of the self-supervised network to perform a classification as either benign or malignant.

The proposed experiments were conducted on an intel i7 CPU desktop with 32 GB of RAM and an NVIDIA GTX 960 graphic processing unit (GPU) with 4 GB of graphic memory. For developing the proposed 3D, CT scan, lung segmentation system, the PyTorch deep learning framework was used with CUDA support and CUDNN library support.

### 3.1. Segmentation Part

Training and testing experiments were performed using the Decathlon dataset. The dataset provides 96 input 3D CT scans. The data were divided into 64 CT scans for training and 32 for network testing. The segmentation’s accuracy directly influences the success or failure of the segmentation process. Therefore, four measurement variables, namely, Dice, sensitivity, specificity, and accuracy, are used. The assessment also depends on true positives (TP), false positives (FP), true negatives (TN), and false negatives (FN).

Dice: a tool for measuring the degree to which predictions and actual results overlap. The better-predicted outcome will have a higher DSC value, which ranges from 0 to 1. In the proposed experiments, the following evaluation metrics can be computed as Equations (11)–(14).

(11) $$\mathrm{Dice}\;=\frac{2\mathrm{TP}}{\;2\mathrm{TP}\;+\;\mathrm{FP}\;+\;\mathrm{FN}}$$

(12) $$\mathrm{Sensitivity}\;=\frac{\mathrm{TP}}{\;\mathrm{TP}\;+\mathrm{FN}}$$

(13) $$\mathrm{Specificity}\;=\frac{\mathrm{TN}}{\;\mathrm{TN}\;+\mathrm{FP}}$$

(14) $$\mathrm{Accuracy}\;=\frac{\left(\mathrm{TN}\;+\;\mathrm{TP}\right)\;}{\left(\mathrm{TN}\;+\;\mathrm{FN}\;+\;\mathrm{FP}\;+\;\mathrm{TP}\right)}$$

During training and testing experiments, different experiment settings were adopted. Table 1 provides all the experiment settings used in the conducted experiments, where TP, TN, FP, and FN stand for true positive, true negative, false positive, and false negative voxels, respectively.

Table 2 provides the results of the segmentation in terms of accuracy and processing speed.

As mentioned in Table 2, the developed lung cancer segmentation system provides encouraging results that outperform the state-of-the-art results on the Decathlon dataset. Aiming to obtain the best segmentation performances, we performed the network training and testing using different optimizers; in this work’s case, AdamW and Nadam. Table 3 reports the obtained performances.

As mentioned in Table 3, by modifying the network optimizer, we succeeded in contributing to better segmentation results. By using the Nadam optimizer, better segmentation performances were obtained. The segmentation accuracy was improved by around 1% compared to the AdamW network optimizer.

As mentioned above, different evaluation metrics were used to highlight the network segmentation performances obtained. The achieved results obtained in the conducted experiments are reported in Table 4.

### 3.2. Classification Part

In the proposed work, we aim to develop a full system for early lung cancer diagnosis. The proposed system consists of two main parts: a segmentation head (detailed in the previous subsection) and a classification head based on the self-supervised network, which will be described in the following.

We should add a classification part for the segmentation results to contribute to a lung cancer diagnosis system. In this work, the output of the segmentation part (segmented CT scan) will be fed into the classification part to be classified as either malignant or benign. Table 5 reports all the experiment settings used during the training process.

The network optimizer plays an essential role in achieving better performances. To this end, we tried two different optimizers: Adam and SGD. Table 6 reports the obtained performances regarding classification accuracy and speed for the two different optimizers.

As depicted in Table 6, the network classification performances were improved when using the Adam optimizer instead of SGD. The best classification rates were achieved with the Adam optimizer.

The number of iterations also highly impacted the network classification performances obtained. Table 7 provides the accuracies obtained when modifying the number of iterations.

As mentioned above, the proposed work presents a full scheme and a full system that can be used for lung cancer diagnosis. Based on the obtained results (segmentation and classification performances), the proposed system presents a new powerful tool that can be used to improve the patient’s life and to combat lung cancer.

Inference time and flops were calculated based on an input with a size of 96 × 96 × 96 based on the sliding window technique. The achieved number of parameters proved that the proposed model is computationally extensive, but a high performance was achieved. Table 8 presents the proposed model’s computation complexity and inference speed.

**Figure 4:**
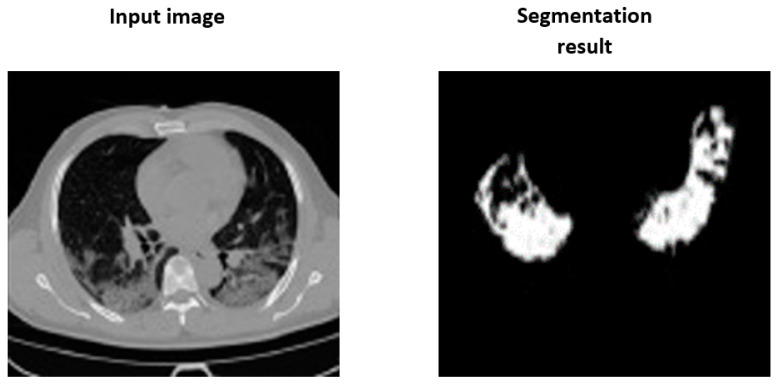
An illustration of the predicted binary mask.

An illustration of the predicted binary mask is presented in Figure 4. The predicted mask highlights the tumor region for further processing, using the classification network to indicate if the tumor is malignant or benign.

### 3.3. Comparison

In order to study the performances obtained by the proposed system and their robustness, a comparison against the state-of-the-art works should be presented. In the proposed work, two main parts for early diagnosis of lung cancer are presented: the segmentation and classification parts. Table 9 presents a comparison against state-of-the-art works on lung cancer segmentation.

In the proposed work, a 3D, CT scan, segmentation system is developed based on the UNETR network. Based on the results mentioned in Table 8, the presented system outperforms the state-of-the-art works.

The proposed lung cancer diagnosis system contains a classification part used to classify the segmentation head output as either benign or malignant CT scans. Table 10 provides an in-depth comparison against state-of-the-art works on lung cancer classification tasks.

The segmentation output is fed into the classification part in order to make a final prediction about the segmentation results. The developed system has shown better performance based on a detailed comparison with state-of-the-art lung cancer classification works. It is extremely important to mention that the proposed classification system works on 3D-input-segmented CT scan images.

## 4. Discussion

### 4.1. Segmentation Part

In order to contribute to a lung cancer segmentation system, different experiments have been conducted to achieve better results. The UNETR network presents a 3D network that can work with 3D input data. In the proposed experiments and the segmentation part of this work, the Decathlon dataset, which provides 3D input CT scan data, is used to train and test the network. We evaluated the impact of modifying the patch resolution; the results are presented in Table 11.

Experiments were conducted to demonstrate that lowering the resolution consistently results in better performance, as presented in Table 10. In particular, reducing the patch resolution from 32 to 16 improves efficiency by around 1.5%. By decreasing the input patch size, the network gains in segmentation performance accuracy, but the processing time will increase.

### 4.2. Classification Part

After the segmentation process, and aiming to achieve better classification rates, in the proposed experiments, we evaluated the self-classification network using two activation functions: ReLU and Leaky ReLU. Table 12 provides all the obtained results in this regard.

Modifying the network activation function from ReLU to Leaky ReLU positively impacted the network classification rate. By using Leaky ReLU instead of ReLU, the classification rate improved by around 1.5%.

The proposed work presents a new tool that can be widely used to diagnose lung cancer and prevent patients from reaching metastases stages. This paper proposes an end-to-end neural network that combines two powerful neural networks: UNETR and a self-supervised network. By combining these two neural networks, the proposed work succeeded in providing a new tool that can be widely employed in CT scan images for combatting lung cancer.

## 5. Conclusions

Lung cancer is one of the most frequent threats to patients’ lives worldwide. Different works have been proposed to address this problem, but none have been efficient, as they either treat the problem of lung cancer through segmentation or classification. Extensive experiments have been conducted to achieve better segmentation and classification results. Aiming to fulfill this goal, in this work, we developed a 3D lung cancer diagnosis system in CT scan imaging. The proposed system consists of two main parts: the first part is for the segmentation, developed on top of the UNETR network, and the second part is used for the classification of the segmentation output, created on top of the self-supervised network.

The proposed system presents a new tool that can be used for 3D, CT scan, lung cancer diagnosis. Very encouraging segmentation and classification results have been obtained, which makes the proposed system efficient enough to help radiologists and doctors in combatting lung cancer. A segmentation accuracy of 97.83% is obtained, and a classification performance of 98.77% is achieved. The main limitations of the proposed model are that it is computationally intensive and requires a high-performance GPU to run smoothly. This limitation can be handled by deploying the proposed model on cloud-based systems or local machines with high capabilities.

## Figures and Tables

**Table 1:** Experiment parameters used.

Parameter	Value
Input patch size	16 × 16 × 16
Learning rate	0.0001
Regression weight	0.00001
Heads number	12
Encoder	VIT-B/16
Optimizer	AdamW/Nadam

**Table 2:** Segmentation performance obtained.

Encoder	Heads	Accuracy	Speed
VIT-B/16	12	97.83	1

**Table 3:** Optimizer’s impact on segmentation performance.

Optimizer	Accuracy
AdamW	96.79
Nadam	97.83

**Table 4:** Evaluation metrics results.

Metric	Value (%)
Dice	96.42
Sensitivity	96.85
Specificity	97.12
Accuracy	97.83

**Table 5:** Experiment settings used.

Parameter	Value
Learning rate	0.01
Momentum	0.6
Optimizer	Adam/SGD
Weight decay	0.00001
Number of iterations	600/800/1000
Batch size	16
Activation function	ReLU/Leaky ReLU

**Table 6:** Optimizer’s impact on classification performance.

Optimizer	Accuracy
SGD	97.18
Adam	98.77

**Table 7:** The number of iterations’ impact on classification performance.

Number of Iterations	600	800	1000
Accuracy	96.47	97.78	98.77

**Table 8:** Computation complexity and inference speed.

Parameters (M)	Flops (G)	Inference (S)
93.32	37.28	10.23

**Table 9:** Segmentation performance comparison.

Method	Imaging Type	Dataset	Sensitivity (%)	Dice (%)	Accuracy (%)
[20]	CT scan	Decathlon	74.28.2	72.55	-
V-NAS-Lung [37]	CT scan	Decathlon	-	66.95	-
3D UNet [38]	CT scan	Decathlon		61.08	-
VNet [39]	CT scan	Decathlon	-	57.82	97.58
[40]	CT scan	Decathlon	-	71.0	-
Proposed	CT scan	Decathlon	96.85	96.42	97.83

**Table 10:** Classification performance comparison.

Method	Imaging Type	Dataset	Accuracy
[41]	CT scan	LIDC-IDRI	91.6%
[42]	CT scan	LIDC	97.3%
[43]	CT scan	LIDC-IDRI	96.69%
[44]	CT scan	LIDC-IDRI	98.2%
Proposed	CT scan	LIDC-IDRI	98.28%

**Table 11:** Patch resolution’s impact on segmentation performance.

Patch Resolution	Accuracy	Speed
32	96.12	15
16	97.83	11

**Table 12:** Activation function’s impact on classification performance.

Activation Function	Recognition Rate
ReLU	97.23
Leaky ReLU	98.77

## Data Availability

The utilized datasets in this study were downloaded from the Kaggle website, and their links are available upon request.
